# The Role of Thyroid Hormones on Skeletal Muscle Thermogenesis

**DOI:** 10.3390/metabo12040336

**Published:** 2022-04-07

**Authors:** Nadia Sawicka-Gutaj, Abikasinee Erampamoorthy, Ariadna Zybek-Kocik, Angelos Kyriacou, Małgorzata Zgorzalewicz-Stachowiak, Agata Czarnywojtek, Marek Ruchała

**Affiliations:** 1 Department of Endocrinology, Metabolism and Internal Medicine, Poznan University of Medical Sciences, 61-701 Poznan, Poland; vaisy110@gmail.com (A.E.); ariadna.zybek@gmail.com (A.Z.-K.); mruchala@ump.edu.pl (M.R.); 2 CEDM, Centre of Endocrinology, Diabetes and Metabolism, Limassol 3075, Cyprus; angelos5@doctors.org.uk; 3 Department of Diabetes, Endocrinology & Obesity Medicine, Salford Royal NHS Foundation & University Teaching Trust, Salford M6 8HD, UK; 4 Medical School, European University of Cyprus, Nicosia 2404, Cyprus; 5 Laboratory of Medical Electrodiagnostics, Department of Health Prophylaxis, University of Medical Sciences, 6 Święcickiego St., 60-781 Poznan, Poland; neuro@ump.edu.pl; 6 Department of Pharmacology, Poznan University of Medical Sciences, 61-701 Poznań, Poland; agata.rat@wp.pl

**Keywords:** thyroid hormones, skeletal muscle, thermogenesis, hypothyroidism, hyperthyroidism, sick euthyroid syndrome

## Abstract

Nowadays obesity becomes a significant global problem. Hence, recently more and more attention has been paid to substances present in the body that have a significant impact on metabolic processes and thermogenesis, in the context of their potential use in the prevention and treatment of obesity. It is well known that the relationship between thyroid hormones and obesity is multilayered, however recently, more and more information about the possible relation between thyroid hormones and muscle metabolism has been published. The aim of this review is to present the most updated information on the physiological impact of thyroid hormones on muscle tissue, as well as pathological changes related to the occurrence of various types of thyroid disorders, including hypothyroidism, hyperthyroidism and sick euthyroid syndrome. However, the data in humans still remains insufficient, and further studies are needed to fully explore the thyroid-muscle cross-talk.

## 1. Introduction

Obesity is an epidemic that has become a significant problem globally. Obese individuals are at an increased risk of various health issues such as diabetes, cardiovascular diseases, fatty liver disease, cancers as well as social challenges [1,2]. The lack of balance between energy input and energy expenditure (EE) is a major contributor to obesity [3]. Modern-day sedentary lifestyle has tipped the scale increasing the rate of obesity worldwide. Weight gain prevention and treatments are being studied extensively with a number of studies emerging to increase EE. Among them, the usage of the body’s regulation of thermogenesis to manipulate EE looks promising [4].

Thermogenesis is the release of heat as a result of metabolic processes of the body. The main source of thermogenesis comes from basal metabolic rate (BMR) which accounts for 60–75% of EE of an adult human [4]. Movement-related thermogenesis and adaptive thermogenesis account for the rest [4]. Few pathways known to be responsible for thermogenesis are B3 adrenergic signalling and its effects on lipogenesis, insulin/IGF1 signalling, and thyroid hormone regulation [5,6]. Brown adipose tissue (BAT) is one of the organs responsible for thermogenesis in humans and is exhibited to create heat through uncoupling of respiration through uncoupling protein 1 (UCP-1) in response to cold, exercise and diet [7,8]. Although BAT thermogenesis is under great research, skeletal muscle thermogenesis has become particularly of interest for the prevention of obesity as it composes ~40% of human body mass thus contributing to a major portion of BMR and thermogenesis in humans [9]. Skeletal muscle can dissipate heat through shivering and non-shivering mechanisms. Heat is produced through shivering by ATP hydrolysis of involuntary repetitive contraction of muscle, but over time causes muscle fatigue [9]. On the other hand, non-shivering thermogenesis (NST) in skeletal muscle could be utilized to prevent and treat obesity.

Thyroid hormones (triiodothyronine (T3) and thyroxine (T4)) regulate the BMR of the human body [10], playing a key role in EE. Weight changes have been attributed to altered levels of thyroid hormones with their use for the treatment of obesity currently being explored [11,12,13]. The relationship between thyroid hormones and obesity is multilayered, with influences on each other and other processes of the body [14]. The link between obesity and the hypothalamic-pituitary-thyroid axis, and the thyroid hormones’ influence on weight through its effects on appetite, lipogenesis, and thermogenesis has also been investigated [15]. Thyroid hormones regulate thermogenesis through various mechanisms such as interactions with adrenergic systems and regulation of uncoupling proteins with studies ranging from animals to humans [16,17]. Recently, Maushart et al. demonstrated that in euthyroid individuals, the level of free T4 affects cold-induced thermogenesis (CIT) as volunteers with higher free T4 levels exhibited a 4 fold increase in CIT than those with lower levels of T4 [18]. Furthermore, thyroid hormones’ influence on thermogenesis spans different systems of the body with their effects on the liver, peripheral blood vessels, sympathetic nervous system, central nervous system, adipose tissue and skeletal muscle [19,20,21]. Thyroid hormones’ significant role in adipose tissue mediated thermogenesis and mechanisms behind this is being researched extensively revealing the involvement of BAT, interactions with the sympathetic nervous system, browning of white adipose tissue, beige adipocytes, mitophagy, selenoprotein biosynthesis, UCP-1 involvement, and mitochondrial biogenesis [21,22,23,24,25,26].

## 2. Thyroid Physiology

### 2.1. Regulation of Sarcoplasmic Reticulum Ca^2+^ ATPase (SERCA)

**Figure 1:**
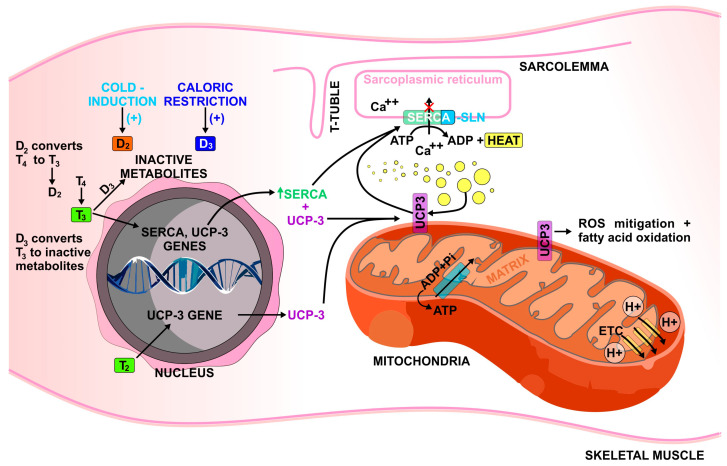
Mechanisms that have been proposed to be responsible for thyroid hormones’ regulation of skeletal muscle thermogenesis. SLN, sarcolipin; D2, type 2 deiodinase; D3, type 3 deiodinase; ADP. Adenosine diphosphate; ATP, adenosine triphosphate, Pi, inorganic phosphate.

The activity of sarcoplasmic reticulum (SR) Ca^2+^—ATPase (SERCA) in skeletal muscle is shown to play a pivotal role in heat generation and thermogenesis [27]. SERCA pumps 2 calcium ions into the lumen of SR using ATP hydrolysis and thus also heat production as a result. It has been demonstrated that SERCA along with its inducer sarcolipin (SLN) in skeletal muscle contributes to NST through SR-Ca^2+^ cycling [28,29]. This increase in energy expenditure through SERCA/SLN regulated NST has been proposed to treat obesity [30]. There is a dependency of T3 in the expression of SERCA 1 in fast-twitch fibres during the postnatal period and an increase in T3 levels is shown to be important for the differentiation of myotubes and SERCA (Figure 1) [31]. Thyroid hormones were shown to significantly regulate SERCA expression in skeletal muscle of animal models [32]. There is an increase in SERCA activity with induction of fast muscle isoform (SERCA1) in slow muscle fibers during high-level T3 states. In addition, T3 downregulates SERCA2a isoforms which are usually expressed far more in slow fibers. The authors suggested that T3 regulates NST through the expression of SERCA1 [32]. Furthermore, the regulation of rabbit skeletal muscle SERCA isoforms by the actions of thyroid hormones has been demonstrated [33]. This study showed an increased expression of SERCA1 while SERCA 2 expression in hyperthyroid red muscle was reduced. In the hypothyroid state, however, there was a decreased expression in SERCA1 of red muscles but no change in SERCA2 expression [33]. This again suggests the contribution of thyroid hormones on skeletal muscle thermogenesis. In a more recent study, thyroid hormone receptor alpha 1 (TRa1) loss-of-function mouse showed an increase in SLN by >5 fold [34]. The authors suggest that the increase in SLN might be a way for skeletal muscle to compensate for the loss of TH function to maintain thermogenesis [34].

### 2.2. Regulation of Mitochondrial Uncoupling Protein 3 (UCP-3)

Another mechanism that has been explored is the role of thyroid hormones in the expression of skeletal muscle uncoupling proteins and the impact of this on thermogenesis. Uncoupling proteins are an important aspect of energy metabolism and are studied for their potential for obesity treatment [35]. Uncoupling protein 1 (UCP-1) plays a major role in BAT-mediated thermogenesis by uncoupling oxidative phosphorylation in mitochondria and dissipating the energy as heat. This mechanism has been demonstrated to be mediated by thyroid hormones [8,36]. In skeletal muscle, UCP-3 which is a homolog of UCP-1 is expressed primarily and is observed to play a role in metabolism and is mediated by thyroid hormones [16,35]. However, the mechanisms of UCP-3 have been shown to be not analogous to UCP-1 in BAT. The main roles of UCP-3 in skeletal muscle are to prevent mitochondrial reactive oxygen species-mediated damage and fatty acid oxidation [35]. Sprague et al. observed the involvement of thyroid hormones and UCP3 on methamphetamine (METH)-induced hyperthermia [37]. This study exhibited thyroid hormones’ role in METH-induced hyperthermia as thyroparathyroidectomized animals elicited a hypothermic response, whereas levothyroxine-supplemented rats restored their hyperthermic functions. This study also demonstrated that UCP3 played a vital role in METH-induced hyperthermia. A follow-up study demonstrated that there is a linear relationship between levels of circulating plasma TH and levels of skeletal muscle UCP3 protein and hyperthermic effects [38]. However, UCP-3′s effects on skeletal muscle thermogenesis are still under debate and its mechanisms are unclear with studies demonstrating contradicting evidence [16,39]. One proposed mechanism of its association to thermogenesis is through its relationship with SERCA/SLN (Figure 1) [9]. Marchi et al. observed that silencing of UCP-3 increased SERCA pumping activity and ATP production in mitochondria but decreased cytosolic Ca^2+^ levels [9,40]. They proposed that UCP-3 negatively affects SERCA activity by limiting ATP synthesis. However, further investigation is required on this topic.

### 2.3. Regulation of Thyroid Hormone Receptor Alpha 1 (TRa1)

Thyroid hormone receptor alpha 1 (TRa1) in skeletal muscle is shown to be vital for the actions of these hormones on skeletal muscle. A recent study used skeletal muscle-specific TRa1 loss-of-function mouse to demonstrate the crucial role of TRa1 receptors in skeletal muscle for the energy expenditure increase by thyroid hormones [34]. The skeletal muscle composition was observed to be changed to oxidative phenotype. In addition, sarcoplin was increased as a possible mechanism to preserve metabolism in these TRa1 loss-of-function mice. It was also shown that there was a small role of these TRa1 receptors on diet-induced obesity. However, the thermogenesis function of these mice remained preserved. These findings illustrate that the TRa1 receptor is not an important factor for thyroid hormone-induced thermogenesis in skeletal muscle and there seems to be a slight dissociation between influences of thyroid hormones on metabolism and thermogenesis. These results were compared with a previous study which demonstrated that global TRa knockout (KO) mice exhibited cold intolerance [41]. The discrepancy between both studies was explained by Nicolaisen et al. by stating that global Tra KO mice are deprived of TRa receptors not only in skeletal muscle but also all over the body, which might be the cause of loss of thermogenesis observed in these TRa KO mice. In fact, TRa1 was shown to be important in the regulation of BAT-mediated thermogenesis [6,36].

### 2.4. 3,5-diiodo-L-Thyronine (T2) Effects

3,5-diiodo-L-thyronine (T2) is an endogenous derivative of thyroid hormones with influences on metabolism and thermogenesis mainly working at the mitochondrial level [42]. In skeletal muscle, T2 was found to cause a change in muscle type from slow to fast-twitch muscle fibers and increase AMPK phosphorylation associated with fatty acid oxidation; an increase in sarcolemma membrane-associated GLUT4 protein was observed as well [43], which mediates the insulin-induced glucose uptake in muscle cells. Furthermore, in skeletal muscle of high fat diet-fed rats, T2 is found to affect aspects of mitochondrial functions and cause a switch of muscle fibers to glycolytic type [44]. In terms of thermogenesis, T2 has been exhibited to improve cold tolerance in hypothyroid rats and shown to influence BAT mediated thermogenesis through its effects on UCP-1, cytochrome c oxidase (COX) activity, and mitochondrial biogenesis [45,46]. Furthermore, in a study by Lombardi et al., the effect of T2 on skeletal muscle thermogenesis was explored, demonstrating a rise in skeletal muscle thermogenesis in T2-injected hypothyroid rats. It was shown that the mechanism responsible for this is mitochondrial uncoupling as proton leak occurs across the inner membrane to the matrix, releasing heat in the process. In addition, free fatty acids (FFA) were shown to be critical in T2-induced mitochondrial uncoupling and thermogenesis in skeletal muscle [47,48]. Furthermore, in diet-induced obese mice models, T2 is demonstrated to enhance mRNA expression of mitochondrial uncoupling protein UCP3 in skeletal muscle (Figure 1) [49].

### 2.5. Role of Deiodinase Enzymes

Type 2 deiodinase (D2) is an enzyme responsible for the activation of pro-hormone T4 into active T3 for further action in tissues whereas type 3 deiodinase (D3) converts T3 and T4 into inactive metabolites [50]. In Thra knockout mice, which is said to act as a model for BAT deficiency, D2 activity was shown to be increased in soleus muscle after 4 h of cold exposure, double the rise of Wild type mice. Furthermore, after 16 h of cold exposure, the D2 activity in Wild type mice returned to normal levels while it increased even more in Thra knockout mice [51]. Another study conducted in rat models demonstrated that D2 is induced in skeletal muscle fibers of these cold-induced rats [52]. There was a 2.3-fold increase in D2 activity in the red soleus muscle, though the increase was higher in BAT (10-fold). However, after 10 days of cold exposure, D2 activity returned to baseline in both BAT and red soleus muscle but saw an increase by 2.8-fold in white glycolytic gastrocnemius muscle. Even though BAT D2 returned to normal, serum and total T3 were shown to be increased, which indicates the importance of D2 and thyroid hormones on fast-twitch skeletal muscle fibers in long-term adaptive thermogenesis [52].

Calonne et al. showed that a decrease in rat model skeletal muscle protein turnover after a considerable amount of weight loss could be responsible for the suppression of thermogenesis which causes catch-up fat deposition [53]. It was also found that T3 was lowered in skeletal muscle during caloric restriction and refeeding period. D2 was also much lower in gastrocnemius muscle during refeeding, however, showed no change in soleus or tibialis anterior. One of their main findings was the markedly increased D3 in skeletal muscle during and after the caloric restriction period [53]. A follow-up study conducted in rat models further exhibited D3′s role in skeletal muscle during catch-up fat gain after caloric restriction, suggesting the possible influence of D3 on suppression of adaptive thermogenesis by the inactivation of thyroid hormones, leading to fat deposition [54]. D3 activity was shown to be lowered during the caloric restriction period and remained at a lower level during catch-up fat deposition. Although blood levels of thyroid hormones were at normal levels, there was local hypothyroidism in skeletal muscle, leading to a decrease in adaptive thermogenesis [54]. This could potentially be a clinical issue and new biomarkers might be required as plasma thyroid hormones are not indicative of tissue-specific thyroid hormone levels.

## 3. Hypothyroidism

Hypothyroidism is a condition of thyroid hormone deficiency and can manifest with various symptoms including lethargy, dry skin, constipation, fatigue as well as cold intolerance and weight gain. Among many organs affected, skeletal muscle is one of them resulting in muscle weakness and cramps [55]. Recently, Zhou et al. demonstrated the cellular mechanisms of hypothyroidism induced skeletal muscle weakness [56]. The study induced hypothyroidism in mouse models through TRa1 mutation and assessed the gastrocnemius muscle. The results of the study showed reduced autophagy in skeletal muscle as well as decreased proteins of mitophagy and factors involved in mitochondrial biogenesis. In addition, there was downregulation of genes related to lipid metabolism and changes in slow and fast-twitch skeletal muscle fibers. In our study conducted in human participants, irisin which is an adipo-myokin is seemed to be affected in thyroid dysfunctions with lower irisin serum levels discovered in hypothyroid participants, most likely due to muscle destruction [57]. We have also demonstrated that irisin levels are low in patients with overt hypothyroidism with the levels being significantly decreased in a long-lasting hypothyroid state [58].

Cold-induced thermogenesis (CIT) is illustrated to be decreased in patients with overt or subclinical hypothyroidism [59]. The influence of the hypothyroid state on skeletal muscle thermogenesis has not been explored in humans. Rats are illustrated to be cold intolerant in the hypothyroid state. However, rats like humans use brown adipose tissue (BAT) and skeletal muscle for thermogenesis which are both affected by thyroid hormones. The effect of hypothyroidism and skeletal muscle thermogenesis specifically was studied using rabbits since rabbits use skeletal muscle as a primary source of thermogenesis [60]. The experiment ensured that there was no BAT recruitment in these rabbit models and focused on skeletal muscle thermogenesis alone. This study demonstrated that hypothyroid rabbits were able to maintain body temperature after 10 days of cold exposure. There was also an observation of increased skeletal muscle mitochondrial oxygen consumption in both normal and hypothyroid rabbits as well as an increased expression of SERCA1 which are suggested to be possible reasons for the cold tolerance of these rabbits. Thus, the authors concluded that skeletal muscle thermogenesis in a cold environment does not solely rely on normal thyroid function. Conversely, mRNA levels of myosin heavy chain fast isoforms and SERCA 1 isoforms were decreased in cold-acclimated hypothyroid zebrafish [61].

One of the major outcomes of hypothyroidism is low metabolic rate and energy expenditure which results in weight gain [10,12,55]. It has been demonstrated that treatment of hypothyroid individuals decreases total body weight and body fat mass with a great relationship between changes in thyroid hormones and body weight [12]. However, animal models have illustrated slightly different outcomes. In a study conducted in 2020, hypothyroid mice with knockout of gene encoding sodium-iodine symporter were studied. They observed that these hypothyroid mice were leaner with reduced body weight than their euthyroid counterparts [62]. This was partly due to less food intake by these mice which is consistent with a previous study that demonstrated that hypothyroid mice’s calorie intake and body fat were decreased [63]. Most importantly, Kaspari et al. observed a significant increase in skeletal muscle thermogenesis in hypothyroid mice with only a moderate increase in BAT regulated thermogenesis although mice were still cold intolerant. They demonstrated that although hypothyroidism did prevent their animal models from maintaining body temperature, adaptive skeletal muscle thermogenesis could be a compensatory mechanism to hypothyroidism-induced cold intolerance in animals to try and regulate their body’s temperature. Furthermore, this study found that there was a significant increase in SLN mRNA and protein levels in the soleus muscle of these hypothyroid mice which also remained high after a cold challenge compared to euthyroid mice. This again clearly indicates the importance of the thyroid hormone’s role in the regulation of SERCA/SLN mediated skeletal muscle thermogenesis. The authors of the study, therefore, suggest that skeletal muscle thermogenesis is a significant factor in terms of TH-induced thermogenesis, particularly through SERCA/SLN regulation.

## 4. Sick Euthyroid Syndrome

Sick euthyroid syndrome which is also known as the non-thyroidal illness, or low T3 syndrome (NTIS), is a transient alteration in thyroid hormone levels despite normal thyroid function among critically ill patients. Generally, low levels of total and free T3 with normal T4 and TSH levels are observed in this condition [64]. A decrease in TH levels during illness is suggested to be the body’s attempt at conserving energy [31]. By decreasing thyroid hormones’ influences on anabolic processes of skeletal muscle, the energy required is thus also lowered during critical illness [31]. In addition to low TH levels, a study on patients with NTIS with septic shock demonstrated that the expression of genes responsible for skeletal muscle function through THs is altered [65]. Furthermore, considering mediators that have been explored to affect skeletal muscle thermogenesis, skeletal muscle TH receptors (TRa1) were shown to be down-regulated in non-septic shock NTIS patients [66]. However, the mRNA levels of SERCA1, SERCA remained unchanged while UCP3 expression was higher. The authors predicted that the increase in UCP3 was likely due to an increase in fatty acids during starvation seen in these patients, which is another condition where UCP3 is typically increased.

In a study by Boelen et al. observing NTIS in mice models, TH metabolism in skeletal muscle had different effects depending on the type of illness [67]. In acute inflammation and bacterial sepsis, TH transport into the cell decreased, while no change was seen in chronic inflammation. In addition, D2 and D3 concentrations differed depending on the state of illness as well. However, in all states, T3 and T4 concentrations were lower in NTIS than in control mice models.

## 5. Exogenous Levothyroxine

Levothyroxine (LT4) is an exogenous thyroid hormone (T4) indicated for the treatment of hypothyroidism [55]. A study conducted in 2019 demonstrated the increase in cold-induced thermogenesis in hypothyroid patients treated with levothyroxine and whose TH levels were restored to euthyroid state [59]. The authors specified that the increase in CIT in these patients could be due to both BAT-induced and skeletal muscle thermogenesis. Levothyroxine’s effect specifically on skeletal muscle thermogenesis has not been studied. However, weight changes with levothyroxine treatment which induced iatrogenic subclinical hyperthyroidism did not elicit long-term weight changes compared to euthyroid patients [68]. In addition, the use of levothyroxine in euthyroid obese patients has been explored by De Geronimo et al. [13]. They concluded that levothyroxine treatment should only be used for hypothyroidism since there is no current evidence to use this hormone for the treatment of obesity in euthyroid individuals. Furthermore, to potentially cause weight loss, supraphysiological doses of levothyroxine are likely needed which will cause other side effects. According to Kyriacou et al., an approach of targeting selective tissues in the body with thyroid hormones can help avoid potential side effects from other organs whilst regulating lipogenesis and weight [69].

## 6. Hyperthyroidism

Hyperthyroidism is a state of higher than normal levels of thyroid hormones with symptoms such as palpitations, restlessness, anxiety, hyperreflexia and most importantly weight loss (in about 90% of patients) and heat intolerance [12,70]. Animal models have been experimented with to show a link between hyperthyroidism and increased skeletal muscle thermogenesis. In a study by Arruda et al., T4-induced hyperthyroid rabbits illustrated an increase in SERCA activity and heat release [71]. There was an 0.8-fold increase in SERCA activity in white muscle while red muscle had a 4-fold increase, suggesting that SERCA activity could be a major contributor to heat generation in hyperthyroidism. Furthermore, Meis et al. pointed out that there is an increase in sarcoplasmic reticulum protein in hyperthyroid rabbits, with a great rise in SERCA 1 subtype in red muscles [72]. They also specified that the changes in red muscle were more significant than white muscle, with red muscles demonstrating more Ca^2+^ in their vesicles and producing more heat. There was a 40-fold increase in heat production by SERCA in red muscle in the hyperthyroid state of rabbits, mainly due to the expression of SERCA1.

Furthermore, skeletal muscle weakness is observed in patients with hyperthyroidism. Hyperthyroidism with these associated muscle symptoms such as skeletal muscle weakness, paralysis, and pain is mainly referred to as thyrotoxic myopathy [73]. Muscle strength and cross-sectional area were reduced in patients with both overt and subclinical hyperthyroidism which improved after treatment [74], while muscle protein breakdown with the increased amino acid release was seen in hyperthyroid women with Graves’ disease [75]. We have observed that normalization of thyroid function in hyperthyroid women leads to weight gain resulting from an increase of fat tissue, while muscle mass remains unaltered [76]. Also, titin and dystrophin- potential biochemical markers of muscle dysfunction were lower in hyperthyroid patients and did not normalize after the therapy, which suggests that some changes of muscle protein are irreversible [77]. In another study, Riis et al. studied the contents of Ca^2+^ ATPase and Na^+^/K^+^ ATPase in skeletal muscle of hyperthyroid patients [78]. In this study, Graves’ disease patient’s muscle biopsies were taken which showed an increase in the regulator of skeletal muscle thermogenesis, Ca^2+^ ATPase. They also observed that the contents of these pumps correlate to plasma thyroid hormone levels and resting EE. In the Maushert et al. study, CIT was shown to not increase in overt hyperthyroid patients as the skin temperatures of both hyperthyroid and euthyroid individuals were not different after mild cold exposure [79]. The authors suggest that according to the data collected, the lack of CIT in these patients is due to high resting EE in a hyperthyroid state, making further thermogenesis during cold exposure needless.

## 7. Summary

Thyroid hormones are strongly associated with skeletal muscle thermogenesis in both the euthyroid and dysthyroid states. The mechanisms underlying thyroid hormones’ effect on skeletal muscle thermogenesis specifically are explored with studies involving SERCA, UCP-3, TRa1, and deiodinase enzymes. Among them, SERCA, UCP-3 and deiodinase enzymes are illustrated to play a role, whereas TRa1 seems to not have an effect. Other than T3 and T4, thyroid hormone metabolite, T2 has also been demonstrated to be playing a part in the process. There is, however, insufficient data in humans and as such further studies are needed. Exogenous thyroid hormones for the treatment of obesity can be further investigated, targeting skeletal muscle thermogenesis, but ideally, they need to be modified to target specific organs, such as the brown adipose tissue and the skeletal muscle.
